# Recommendations for managing adult acne and adolescent acne based on an epidemiological study conducted in China

**DOI:** 10.1038/s41598-024-67215-2

**Published:** 2024-07-15

**Authors:** Yan-ting Liu, Ya-wen Wang, Chen Tu, Jian-wen Ren, Jia Huo, Xiao-juan Nan, Jia-hao Dou, Zi-he Peng, Wei-hui Zeng

**Affiliations:** 1 Department of Dermatology, The Second Affiliated Hospital of Xi’an Jiaotong University, Xi’an, 710004 Shaanxi China; 2 Health Science Center, Xi’an Jiaotong University, Xi’an, 710004 Shaanxi China

**Keywords:** Acne, Adult, Adolescent, Epidemiology, Diseases, Medical research, Risk factors

## Abstract

There are numerous differences between adult acne and adolescent acne in terms of causes, distribution, and characteristics of skin lesions, as well as treatment. This paper aims to summarize the differences between adult and adolescent acne in China, in order to propose more suitable ways to improve their quality of life. We collected basic information, acne-related information, acne-affecting factors, quality of life scores and treatment-related information of acne patients. A total of 552 questionnaires were collected. Adult acne is typically predominant on the cheeks, similar to adolescent acne, with a relatively lower incidence in other areas, apart from the jawline. Pigmentation and depressed scars are present in nearly half of acne patients, while hypertrophic scars are less frequently observed. Teenagers often have a higher consumption of dairy products, sugary drinks, and high-sugar and high-fat foods. Eczema is more common in adult acne. Additionally, more adults than teenagers experience stress and poor quality of life related to acne. Adolescents are more likely to seek treatment online and on social media. Clinicians must thoroughly evaluate diverse risk factors and formulate personalized acne management strategies for patients with different types of acne.

## Introduction

Acne is a prevalent skin disease worldwide, accounting for 9.4% according to the Global Burden of Disease (GBD) study^1^, and is considered the eighth most common disease worldwide^2^. In China, the prevalence of acne in 2019 was considerably lower, with a prevalence of 4.1%, as reported by the GBD study^3^. Acne is a chronic inflammatory dermatosis characterized by the presence of comedones, inflammatory papules, pustules, nodules, cysts, and scars^2,4,5^. Although acne can affect people of all ages^5,6^, it is commonly associated with adolescence due to its high incidence during this period^4^. Researchers have found that adolescents and adults with acne exhibit unique epidemiological and treatment characteristics^7^. Among them, acne with an onset age of ≤ 25 is labeled as adolescent acne, whereas acne with an onset age of > 25 is identified as adult acne^6^. Medical professionals have conducted comprehensive studies on adult acne in recent years, dividing it into two subtypes: persistent adult acne and late-onset adult acne.

Presently, no study has comprehensively compared and analyzed the epidemiological characteristics of adult and adolescent acne within the Chinese acne population, and new potential acne risk factors have emerged in recent years. Therefore, this study aims to explore the differences in clinical characteristics, risk factors, and the impact of acne on the lives of adult and adolescent acne patients in China, in order to identify potential triggers and provide corresponding lifestyle improvement suggestions for adult and adolescent acne patients in China.

## Method

From March 2023 to April 2023, we conducted an observational cross-sectional study in the Dermatology outpatient department of the Second Affiliated Hospital of Xi’an Jiaotong University. We distributed questionnaires to acne patients attending the outpatient department. Our questionnaire focused on four aspects: general patient information, acne-related information, acne influencing factors, quality of life evaluations and treatment-related information. The general information survey included basic patient information, such as gender, age, Body Mass Index (BMI), and occupation. We asked about when the patients first developed acne, how long they had the disease, how severe their acne was (evaluated by Pillsbury’s 4-stage grading method), whether they had acne scars and where on their body the acne was located. Inquiring about the influencing factors of acne, we included aspects such as family history, diet, sleep, and mood that may affect or worsen acne. We used the Dermatology Life Quality Index (DLQI) to evaluate the impact of acne on the patients’ quality of life. We chose an online questionnaire platform called “Sojump” to conduct the survey. We utilized IBM SPSS Statistics software (SPSS) version 22 to perform various statistical tests, including the Pearson Chi-square test, Fisher’s exact test, and Wilcoxon rank-sum test, in order to examine potential significant disparities between adult acne and adolescent acne. Results were presented as proportions and percentages. This study received an ethical review exemption from the Ethics Committee of the Second Affiliated Hospital of Xi’an Jiaotong University due to the anonymized collection of patient information, following the principles of the Declaration of Helsinki. Informed consent was acquired from every participant, and strict confidentiality measures were maintained for all gathered data throughout the research.

## Result

### Demographic characteristic

The study investigated 552 Chinese acne patients, with a male to female ratio of approximately 1:2.3 and an average age of 23.2 years. The sample was composed of 387 adolescent and 165 adult patients with acne. Female patients accounted for a greater proportion of adult acne (80.0%) than adolescent acne (64.9%). Our study found that acne most commonly onset between the ages of 13 and 25 years old, with a peak onset age of 18 to 25 years old. The age group most affected by adult acne mainly falls between the ages of 26 and 30 (72.1%), whereas teenagers fall primarily within the range of 18–25 years old (86%). The majority of patients (58.5%) had normal BMI levels (18.5–23.9). Adults with acne had a higher normal BMI proportion (64.3%) than adolescents (55.80%). College students accounted for the majority of adolescent acne sufferers (52.7%), while office workers comprised the majority of adult acne sufferers (80.6%) (Table 1).

**Table 1:** Demographic characteristics of adult acne and adolescent acne patients.

Characteristic	Acne type		p-value^2^
	Adult acne, N = 165^1^	Adolescent acne, N = 387^1^	
Gender			< 0.001
Male	33 (20%)	136 (35%)	
Female	132 (80%)	251 (65%)	
Age group			< 0.001
< 18 years	0 (0%)	56 (14%)	
18–25 years	0 (0%)	331 (86%)	
26–30 years	119 (72%)	0 (0%)	
31–40 years	42 (25%)	0 (0%)	
41–50 years	4 (2.4%)	0 (0%)	
BMI			0.007
Median (IQR)	20.8 (19.5, 23.9)	20.3 (18.4, 23.1)	
Occupation			
Elementary and middle school student	0 (0%)	55 (14%)	
Undergraduate student	5 (3.0%)	204 (53%)	
Graduate student	9 (5.5%)	26 (6.7%)	
Office worker	133 (81%)	87 (22%)	
Freelancer	7 (4.2%)	5 (1.3%)	
Stay-at-home mom	10 (6.1%)	0 (0%)	
Other occupations	1 (0.6%)	10 (2.6%)	
Onset time			< 0.001
8–12 years	3 (1.8%)	34 (8.8%)	
13–15 years	13 (7.9%)	118 (30%)	
16–18 years	37 (22%)	125 (32%)	
18–25 years	69 (42%)	103 (27%)	
> 25 years	43 (26%)	7 (1.8%)	
Duration			< 0.001
< 1 year	31 (19%)	56 (14%)	
1–3 years	42 (25%)	159 (41%)	
3–5 years	25 (15%)	111 (29%)	
5–10 years	48 (29%)	56 (14%)	
> 10 years	19 (12%)	5 (1.3%)	
Severity			0.842
Grade 1	74 (45%)	170 (44%)	
Grade 2	60 (36%)	132 (34%)	
Grade 3	18 (11%)	52 (13%)	
Grade 4	13 (7.9%)	33 (8.5%)	
Pigmentation and scarring			0.857
Only red pigmentation	76 (46%)	176 (45%)	
Pigmentation and depressed scars	66 (40%)	151 (39%)	
Pigmentation and hypertrophic scars	5 (3.0%)	18 (4.7%)	
Pigmentation, depressed and hypertrophic scars	18 (11%)	42 (11%)	
Family history			0.039
No	106 (64%)	246 (64%)	
At least one parent has	25 (15%)	89 (23%)	
At least one sibling has	25 (15%)	33 (8.5%)	
At least one parent and at least one sibling	9 (5.5%)	19 (4.9%)	

^1^n (%).

^2^Pearson’s Chi-squared test; Fisher’s exact test; Wilcoxon rank sum test.

### Clinical characteristic

**Figure 1:**
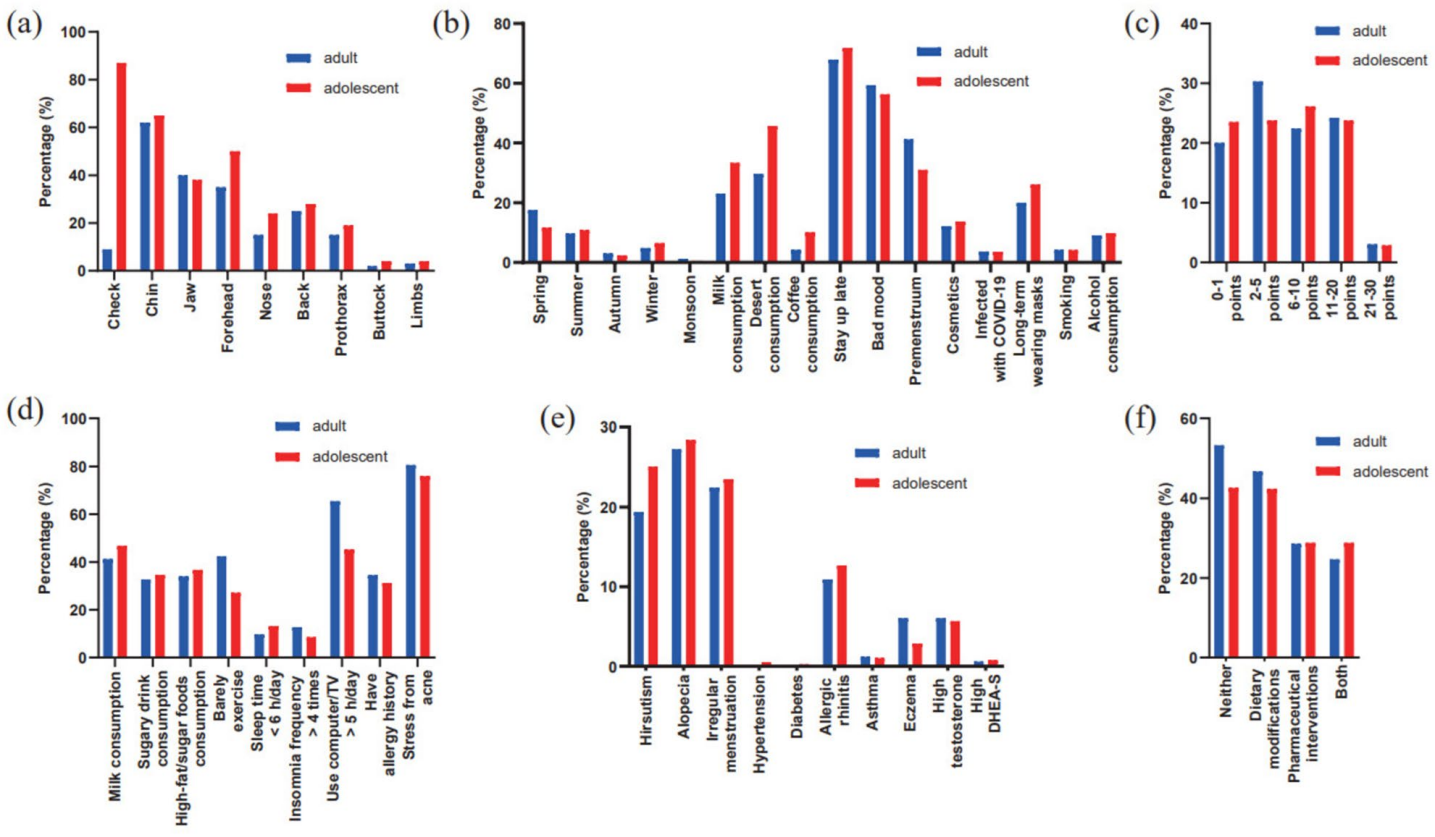
Bar graphs comparing adult acne and adolescent acne in various aspects. (**a**) Comparison of areas affected by acne. (**b**) Comparison of the percentage of patients experiencing worsened acne symptoms due to different risk factors. (**c**) Comparison of scores on the Dermatology Life Quality Index (DLQI). (**d**) Comparison of lifestyle factors. (**e**) Comparison of the proportion of other diseases occurring. (**f**) Comparison of treatment-related information. *PCOS* polycystic ovary syndrome, *DHEA-S* dehydroepiandrosterone sulfate.

Adult acne onset (≥ 16 years) was later than adolescent acne (mean 15.5 years). A higher proportion of adolescents (69.8%) with acne had been affected for 1–5 years compared to adults (40.6%), while the majority of patients affected for over 5 years were adults (Table 1). Acne mainly appeared on the cheeks, chin, jaw, and forehead, while, among adults, the jaw is the sole region with a higher prevalence compared to teenagers. Additionally, acne was found on the back, nose, front chest, buttocks, and limbs, and adolescent acne patients had a higher prevalence on the trunk than adult acne (Fig. 1a). Acne severity ranged mainly from grade I to II, with a higher incidence of pigmentation and depressed scars, while that of hypertrophic scars was lower. At least one close relative of 35.8% of adult acne patients has a history of acne. Similar results are also observed among adolescent acne patients (36.4%) (Table 1).

### Aggravating factors

33.2% of the patients reported seasonal aggravation, which was particularly frequent in spring and summer and affected adult acne more significantly. Acne patients are particularly vulnerable to various factors, including staying up late, emotional stress, consumption of milk and desserts, and the premenstrual phase. Interestingly, staying up late, consuming milk, desserts and coffee, and experiencing worsening of acne due to prolonged mask-wearing are more prominent in adolescents, whereas the manifestation of acne triggered by negative emotions and the premenstrual period is more apparent in adults. Additionally, other factors like cosmetics, smoking, alcohol consumption, and COVID-19 infection have been identified to exacerbate acne, yet there are negligible discrepancies between adolescent and adult acne (Fig. 1b).

Adolescents had a slightly higher total dairy consumption, with yogurt and whole milk being the most frequent types. Adolescents with acne were roughly 2% more inclined than adults to consume sugary drinks and high-sugar, high-fat foods more than thrice weekly. Adolescents with acne indicated roughly 3% more sleep deprivation rates than adults, while the prevalence of insomnia was 4% lower than that in adult acne. Adult acne had a higher incidence of little exercise than adolescents. More than half of the patients (51.3%) spent more than 5 h a day watching TV or using the computer, with the majority of them being adult acne. A history of allergies was slightly more prevalent in adult acne than in adolescent acne. Acne is more likely to cause stress in adults (Fig. 1d).

Compared to adolescents (69.0%), the proportion of adult female patients (81.3%) experiencing hirsutism, hair loss, and premenstrual exacerbation increases. The most common affected areas with hirsutism, hair loss, and premenstrual exacerbation are the cheeks, chin, forehead, and jaw area. In addition to hirsutism and allergic rhinitis, which are more frequently seen in adolescent acne, adults with acne tend to have a higher prevalence of eczema. Two cases of hypertension and one case of diabetes were discovered among adolescent acne patients. 14.3% of acne patients presented abnormal sex hormones, primarily increased testosterone and dehydroepiandrosterone sulfate (DHEA-S), but with no notable differences between adolescent and adult acne (Fig. 1e).

### Quality of life assessment and treatment-related information

Adults are more likely to experience stress as a result of acne, however, as per the DLQI scoring standard, their quality of life is affected to a lesser degree than teenagers (Fig. 1c). Adolescents with acne are inclined to pursue remedial measures on the internet or social media prior to seeking treatment in hospital, and in contrast to pharmaceutical interventions, dietary modifications on the internet are generally received more positively by patients (Fig. 1f).

## Discussion

Gender differences in acne prevalence have been widely noted, with females typically having a higher proportion of acne sufferers than males^3^. Our study supports this trend and also found that the proportion of women with acne was significantly higher among adults than adolescents. However, a meta-analysis conducted in mainland China found that the prevalence of acne was actually lower for females than males^8^. This is consistent with the results of Schafer, Adityan and Thappa et al.^9,10^. A community study conducted in six Chinese cities further found that the prevalence of acne was higher in females than males among adolescents, higher in males in their teens and 20s, and higher in females over the age of 30^11^. This is similar to our results, and also indicates that acne patients of different genders may be affected by different internal hormones and external influencing factors at different ages, leading to gender differences in the prevalence of acne. Acne is a common dermatosis that can affect individuals of all ages^5,6^, with its prevalence peaking during adolescence, affecting 85% of adolescents aged 12–24 years before gradually declining^4^. Our study found that acne most commonly onset between the ages of 13 and 25 years old, with a peak onset age of 18–25 years old. This supports the prevailing view that acne is a disease of adolescence, although adult acne should not be ignored. Researchers have found that the epidemiological characteristics and treatment of adolescent and adult acne differ^7^. For example, the factors that exacerbate acne differ between two distinct populations. Adolescents are more susceptible to the influence of dietary factors, while adult women are more likely to experience hormonal imbalances. Consequently, it is vital to evade potential exacerbating factors, and treatment should consider diverse skin types, lesion location, severity, and characteristics.

The relationship between BMI and acne remains controversial despite research indicating that BMI is among the contributing factors to the development of acne. A possible inverse, dose-dependent relationship between BMI and acne was found in both Israeli adolescents and Taiwanese adult women^12,13^. Conversely, it has been suggested that a lower BMI protects against acne, especially in men^14,15^, due to the association between obesity in males, hyperlipidemia, and severe acne^16^. Our research did not find a correlation between BMI and acne, possibly due to the limited sample size, subjectivity of patient questionnaire responses, and other confounding variables.

Acne vulgaris affects several areas such as the cheeks, forehead, chin, nose, chest, and back^17^. Our findings regarding the distribution of acne on adults and adolescents align with previous studies, where adolescent acne predominantly appeared on the cheeks and trunk^7^. Acne occurring solely on the trunk has been observed in adolescent fitness enthusiasts who consume whey protein as a dietary supplement, based on a series of case reports. Consequently, apart from prioritizing facial acne, it is imperative to enhance care and treatment for trunk acne^18^. Moreover, in adults, acne typically affects the lower third of the face (chin, perioral area, and mandibular line), neck and is less likely to affect the trunk area^6,19^.

Most acne cases manifest as mild acne, with severe acne being rare^14^, and post-acne hyperpigmentation being more commonly observed^10^. Adult acne lesions predominantly present as mild inflammatory papules with an underlying inflammatory nature^6,7^. Adolescent acne typically starts with comedones, progressing from mild inflammatory lesions to severe nodular cystic lesions^4,7^. Adult acne is more prone to scarring compared to adolescent acne, likely attributing to the presence of inflammatory lesions and treatment resistance, which amplify the scarring risk^7^. No significant differences were observed in the severity and scarring formation between adult and adolescent acne, suggesting that the type and severity of acne scarring may depend on factors such as the patient’s age, gender, acne site, acne grading, and duration before treatment^14,20^. Our findings on family history of acne were consistent with previous studies, which found an increased risk of acne in first-degree relatives, such as parents or siblings with a history of acne.

The meta-analysis revealed a significant increase in the incidence of acne among individuals who consumed milk^15^. Among milk consumers, those who consumed skim milk exhibited a stronger association with acne compared to those who consumed low-fat or whole milk^15,21^. Our findings support the idea that dairy consumption may be a potential risk factor for acne, especially among adolescents. In our study, individuals with acne consumed a higher quantity of dairy products, including yogurt and whole milk. Additionally, the consumption of sugary drinks elevates the risk of moderate to severe acne in both teenagers and adults^22,23^. Numerous studies have linked high-fat and high-sugar dietary patterns to acne^23,24^. Specifically, ice cream, cheese, and chocolate have been identified as potential culprits in increasing the risk of acne^25^. We observed significantly higher levels of serum IGF-1 in acne patients^26^, along with increased expression in the epidermis and sebaceous glands^27^. These elevated IGF-1 levels could lead to increased lipogenesis^28^. The hormone content, specifically alpha lactalbumin and branched-chain amino acids (BCAAs), present in milk may induce an increase in IGF-1, thereby potentially causing acne^21^. This could be attributed to the fact that dairy products and sweets increase the levels of insulin-like growth factor-1 (IGF-1), which subsequently amplifies androgen-mediated sebum production^29^. Conversely, regular consumption of fish, fruits, vegetables, and adherence to the Mediterranean diet have shown to be protective factors against acne, possibly due to the antioxidant properties inherent in these foods^15,19,24,30^. Furthermore, our investigation findings indicated that adolescents with acne tend to consume higher quantities of milk, desserts, and coffee in comparison to adult patients. However, the proportion of intake of dairy products, sugary beverages, and high-fat foods is comparable. Hence, it is recommended that teenagers with acne decrease their consumption or even abstain from these types of foods. Furthermore, it is advised that all individuals with acne decrease their consumption of high-fat and high-carbohydrate foods, while aiming for a nutritionally well-rounded and proportionate healthy diet.

A study conducted in China identified a correlation between smoking, alcohol consumption, and the occurrence of teenage acne^11^. Furthermore, similar findings are observed in adult acne^9^. Yang et al. proposed that smoking may contribute to acne development by promoting oxidative stress, leading to the accumulation of lipid peroxides (LPO) in acne. This proposition is strengthened by the notably elevated levels of IL-1α and LPO observed in smoking patients with acne^31^. Our findings indicate that smoking and consuming alcohol can worsen acne symptoms. This phenomenon is observed in both adolescents and adults who have acne. There is no significant statistical difference in smoking and alcohol consumption between adult acne and adolescent acne. Therefore, both teenage and adult acne patients should make efforts to quit smoking and drinking, adopt healthy lifestyle habits, and prevent worsening of acne due to smoking and alcohol consumption.

During the COVID-19 outbreak in China, the study identified that 29.9% of patients experienced worsening of acne symptoms, attributed to a combination of factors^32^. Our research indicate that the incidence of acne caused by COVID-19 is approximately equal among both adolescents and adults. Conversely, the proportion of acne triggered by wearing masks for COVID-19 differs between adolescents and adults, with adolescents having a higher proportion than adults. Findings from a cross-sectional study conducted during the COVID-19 outbreak in China revealed a significant association between prolonged mask wearing (> 28 h per week) and the deterioration of acne symptoms. Moreover, the study indicated that the severity of acne symptoms remained unaffected by the type of mask used^32^. The wearing of masks can exacerbate acne by impairing the skin barrier and obstructing the sebaceous ducts of hair follicles through various means such as mechanical friction, local pressure, increased temperature, and humidity. Furthermore, these potential mechanisms substantiate a dose–effect relationship between the duration, frequency, and the occurrence of acne associated with mask wearing^32,33^. Hence, there are notable distinctions in the management of mask-induced acne during the COVID-19 pandemic when compared to common acne. Additionally, it is crucial to prevent the aggravation of acne in teenagers resulting from mask-wearing. First, it is advisable to use gentle facial cleansers containing antibacterial ingredients to preserve a healthy skin microbiome. Second, it is important to maintain adequate skin hydration to decrease transepidermal water loss. However, traditional moisturizers containing lactic acid and urea should be avoided as they can react with sweat and disrupt the skin’s pH, potentially causing contact dermatitis. Lastly, using masks made from fabrics with a smooth surface can help reduce friction on the skin^34^.

An intriguing study revealed that in the context of the COVID-19 pandemic, the side of the face predominantly exposed to mobile phones during conversation had a higher incidence of acne lesions and/or more severe disease severity^35^. This phenomenon could be attributed to the heightened proliferation of Staphylococcus aureus resulting from the exposure to short-wavelength visible light emitted by smartphones and tablets^36^. Furthermore, the use of computers and TVs was significantly linked to the manifestation of acne^14^, the mechanism underlying acne development may be analogous to that of mobile phones. We also found that using computers or watching TV for more than 5 h per day may be a risk factor for acne, especially in adult acne. This could be attributed to the fact that teenagers have limited access to computers and TVs as their usage is regulated by their parents, whereas adults possess greater autonomy and often require their usage for professional purposes. It is advisable for adult individuals with acne to extensively reduce their utilization of computers, televisions, and mobile phones, particularly outside of work-related obligations.

Asthma, allergic rhinitis, eczema, and other atopic diseases are considered to be risk factors for acne^14^. Strong risk factors for acne include familial hypercholesterolemia, diabetes, and hypertension^30^. Additionally, we observed that the aforementioned risk factors not only exacerbate acne, but also unexpectedly found two cases of hypertension and one case of diabetes among adolescent acne patients. Though the number of cases was small, it is worthy of our attention. Hyperandrogenic clinical features are present in over one-third of adult females with acne^6^. According to a study on endocrine parameters in adult women, 19% of females with acne over the age of 17 were diagnosed with polycystic ovary syndrome (PCOS)^37^. Furthermore, we not only found a higher proportion of adult female acne patients experiencing hirsutism, hair loss, and worsening before menstruation compared to adolescents, but also discovered that the affected areas for these patients are mainly the lower third of the face.

Females with acne displayed significantly higher serum levels of DHEA-S and testosterone compared to those without acne, while there was no significant difference in serum androgen levels among males with and without acne^16,38^. Premenstrual episodes are considered to be a risk factor for moderate to severe acne in females^39^, accounting for 57.7% of recorded episodes^10^. The presence of acne alongside hirsutism, alopecia, PCOS, and nulliparity in adult females provides evidence of a connection between adult acne and hormonal factors like hyperandrogenism^19,40^. No disparity in sex hormone abnormalities was observed between adult and adolescent women with acne. Consequently, hormonal examination and therapy are recommended for women with acne^37,41^. After testosterone is produced by the adrenal cortex and ovaries, most of it binds to sex hormone-binding globulin in the blood serum. Under the action of 5-alpha reductase, it is converted into a more active form, dihydrotestosterone. Testosterone and dihydrotestosterone form androgen receptor (AR) complexes in target cells, which stimulate the secretion and proliferation of lipid cells. Therefore, antiandrogens play a significant role in the treatment of acne, mainly including 5-alpha reductase inhibitors, AR antagonists, and testosterone synthesis inhibitors^42^.

Our investigation supports previous findings suggesting that working individuals have a higher acne risk when compared to freelancers, unemployed individuals, or housewives^19^. This might be due to the work group feeling greater pressure, putting the body in a state of stress. The hypothalamus and cerebral cortex perceive the stress, and through neural transmission, make the burning sensation and itching more pronounced, leading to aggravation of skin inflammation, causing a cycle of stress-itching-inflammation^43^. Therefore, for patients with acne, being in a state of physical and mental pleasure and relaxation is particularly important. Avoiding various inducements of negative emotions and adjusting psychological state are prerequisites for effective treatment methods.

Adult acne is a condition characterized by both medical and psychosomatic features. Additionally, adults with acne vulgaris demonstrate a higher prevalence of neuroticism and common psychiatric disorders compared to the general population^44^. There exists a close bidirectional relationship between stress and acne^45^. Various mechanisms suggest that stress can exacerbate acne^46^. A study involving students revealed increased acne severity and elevated stress ratings during exams. Moreover, a significant association was found between increased acne severity and higher stress levels^47^. Adult women who experience high psychological stress are also prone to acne^19^. Conversely, individuals with acne had notably higher scores for anxiety and depression compared to the control group^45^. Furthermore, the study conducted by Lim and Đurović et al. reported a substantial impact of acne on the sufferer’s quality of life. The average score on the Dermatology Life Quality Index (DLQI) was approximately 4.3, and the incidence of impaired quality of life was significantly higher among females. These findings solidify the notion that acne intensifies the psychological burden on individuals affected by it^48,49^. Our research also identified a reciprocal relationship. Moreover, we found that adults are more prone to experiencing stress related to acne. Conversely, adolescents with acne are significantly more affected compared to adults. It is recommended that acne patients seek medical assistance to treat their condition and ameliorate the negative repercussions arising from it. Adult acne patients can decrease acne exacerbation resulting from stress by enhancing work efficiency or engaging in recreational activities. Adolescents suffering from acne can alleviate the negative impact on their quality of life by engaging in communication with family and friends or seeking professional psychological counseling.

Due to the easy accessibility of online information, an increasing number of individuals with acne are inclined to search for acne treatment options online. Nevertheless, online information originates from diverse sources, and its quality varies. Consequently, there is uncertainty regarding whether the information provided to patients will yield favorable outcomes or exacerbate their acne condition. Online “cures” range from vegetarianism to purported “miracle cures,” yet their efficacy lacks definitive evidence, and certain recommended supplements may potentially provoke or aggravate acne^50^. Yousaf et al. reported that 81% of social media users attempted over-the-counter products, and 40% experimented with dietary modifications, yet a mere 7% of patients noted substantial improvement^51^. Comparable findings were encountered in our investigation, as adolescent acne patients (57.4%) displayed a greater tendency to seek support online or via social media platforms compared to their adult counterparts (46.7%). Moreover, 38.6% of all acne patients pursued dietary modifications, while 30.6% resorted to medication usage. Nonetheless, the questionnaires exclusively obtained from patients attending dermatology clinics further reiterate the lack of effectiveness associated with internet and social media-based treatment programs. Therefore, online information cannot be considered entirely reliable. Hence, patients are encouraged to approach online information with caution, especially adolescents, seek timely medical consultation, and diligently adhere to their physician’s prescribed treatment plan for optimal therapeutic outcomes.

The study has several limitations. Firstly, the sample size is small because the questionnaires were collected from the dermatology outpatient clinic. Secondly, acne is solely self-assessed by the patients, which may not accurately reflect the actual condition. Our innovation lies in conducting a comprehensive investigation of epidemiological data differences between adult and adolescent acne patients in the Chinese population. This allows us to provide specific recommendations for patients.

## Conclusion

In recent years, adult acne and adolescent acne differ in terms of clinical characteristics, risk factors, impact on quality of life, and approaches to seeking treatment. Therefore, we recommend that adolescent acne patients reduce or avoid the consumption of dairy products, desserts, and coffee as much as possible, reduce the time and frequency of wearing masks, openly communicate with family and friends or seek professional psychological counseling to alleviate the negative impact of acne on their quality of life. In addition, adolescent acne patients should utilize the internet cautiously, seek timely professional medical advice, and prioritize treating acne on the face and body simultaneously. Conversely, adult acne patients should be mindful of staying up late, experiencing negative emotions, and premenstrual periods, while actively reducing the use of computers, televisions, and mobile phones, and engaging in regular physical exercise. Furthermore, adult acne patients can reduce acne exacerbated due to stress by improving work efficiency or participating in recreational activities. Lastly, for all acne patients, we recommend reducing the intake of high-fat and high-sugar foods, quitting smoking and drinking alcohol, and maintaining both physical and mental well-being. Furthermore, we recommend hormone testing and treatment for female acne patients, irrespective of whether they are adolescents or adults.

## Data Availability

The datasets generated during and/or analysed during the current study are available from the corresponding author on reasonable request.
